# Metabolomics and pathway analyses to characterize metabolic alterations in pregnant dairy cows on D 17 and D 45 after AI

**DOI:** 10.1038/s41598-018-23983-2

**Published:** 2018-04-13

**Authors:** Y. S. Guo, J. Z. Tao

**Affiliations:** School of Agriculture, Ningxia University, Yinchuan, China

## Abstract

Nutrient flow to the embryo and placenta is crucial for proper development and growth during pregnancy. In this study, a metabonomic analysis was undertaken to better understand global changes in pregnant dairy cows on D 17 and D 45 after timed artificial insemination (AI). Metabolic changes in the blood plasma of pregnant dairy cows were investigated using HPLC-MS and a multivariate statistical analysis. Changes in metabolic networks were established using the MetPA method. Alterations in six metabolic pathways were found on D 17 and D 45, including variations in the level of alpha-linolenic acid metabolism, glycerophospholipid metabolism, pentose and glucuronate interconversions, glycerolipid metabolism, folate biosynthesis, and tyrosine metabolism. In addition to these pathways, 9 metabolic pathways were markedly altered on D 45. These pathways included changes in the one-carbon pool caused by folate; phenylalanine, tyrosine and tryptophan biosynthesis; thiamine metabolism; pantothenate and CoA biosynthesis; purine metabolism; inositol phosphate metabolism; amino sugar and nucleotide sugar metabolism; pentose phosphate; and the TCA pathway. The combination of metabonomics and network methods used in this study generated rich biochemical insight into possible biological modules related to early pregnancy in dairy cows.

## Introduction

Once fertilization has occurred, the fate of a successful pregnancy is determined by the survival of the embryo and foetus. Nutrients are essential for embryo and foetus development, although progesterone plays a vital role in regulating uterine function and embryo development^1^ as well as increasing pregnancy rates based on the increase of its concentrations^2,3^. For example, amino acids (AA) are important components of maternally derived secretions that are crucial for embryonic survival before implantation^4^. Certain maternal metabolism pathways change due to the nutrient transport during early pregnancy. However, the related information on maternal comprehensive metabolic response to early pregnancy remains limited. Hence, global changes in metabolites require characterization.

Metabonomics is a valuable emerging tool to measure the dynamic metabolic response of living systems to stimuli or modification^5,6^ and has been increasingly used to evaluate metabolic changes in dairy cows^7–9^. There have been no previous reports of the use of metabonomics in the study of dairy cows during early pregnancy. Accordingly, this study was designed to provide an evaluation and temporal comparison of the plasmatic metabolome of pregnant dairy cows on D 0, D 17 and D 45 after artificial insemination (AI). HPLC-QTOF/MS was used in combination with pattern recognition methods and pathway analysis methods to look for variation in the metabolic phenotype and to generate a better understanding of the metabolic mechanisms occurring in pregnant dairy cows.

## Results

### Metabolic profiles of Groups A, B, and C

**Figure 1:**
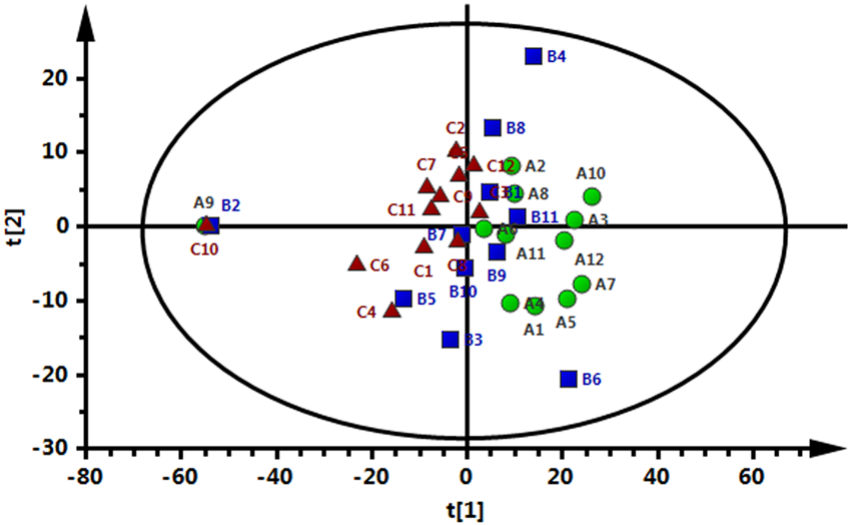
PCA score plot of Groups A (green circles), B (blue squares) and C (red triangles).

**Figure 2:**
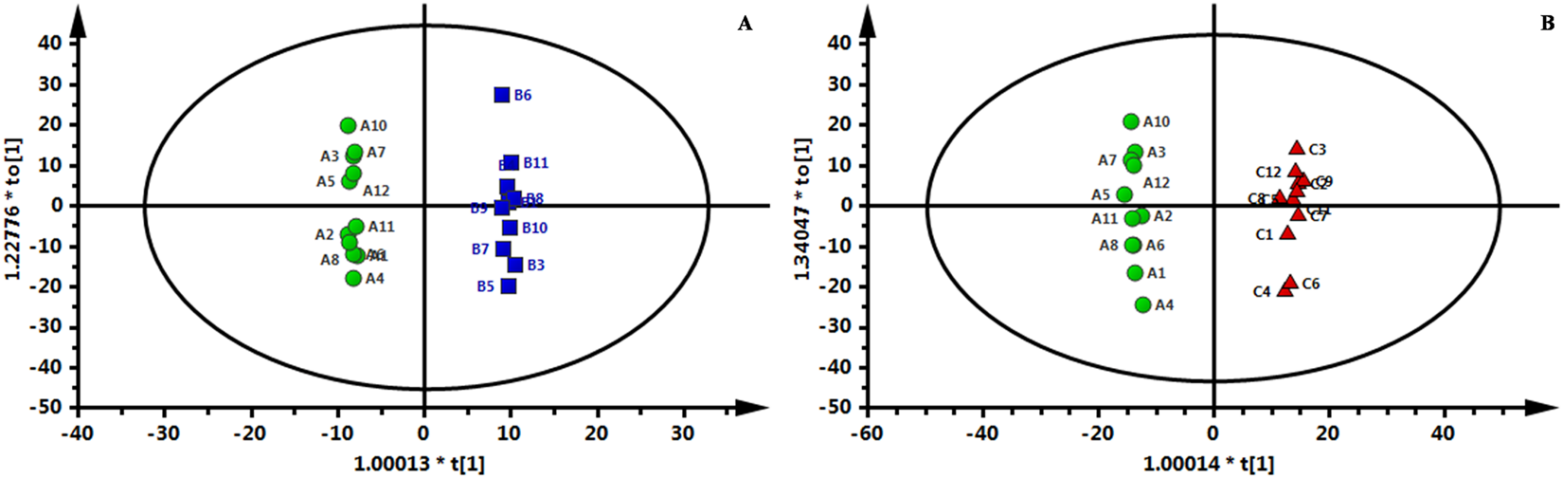
OPLS-DA score plot. (**A**) Group A (green circles) vs. Group B (blue squares); (**B**) Group A (green circles) vs. Group C (red triangles).

Representative HPLC-QTOF/MS ES^+^ and ES^-^ chromatograms are shown in Supplemental Fig. 1A,B. The final data table contained 848 variables (chromatographic peaks). The similarities and differences among Groups A (green circles), B (blue squares), and C (red triangles) are displayed in the score plots of the principal component analysis (PCA) (Fig. 1). The OPLS-DA models indicated clear separations between Group A (green circles) and Group B (blue squares) (R^2^X = 0.57, R^2^Y = 0.996, Q^2^ = 0.752; Fig. 2A) as well as between Group A (green circles) and Group C (red triangles) (R^2^X = 0.571, R^2^Y = 0.995, Q^2^ = 0.889; Fig. 2B). However, Groups B and C were not separated by the OPLS-DA model.

### Discovery and identification of the metabolic markers

The identification of potential metabolic markers was based on their contribution to the variations and correlation within the dataset. According to the OPLS-DA model, Groups A and B had 57 and Groups A and C had 273 (including the 57 for Groups A and B) significantly altered plasma features, with a variable importance in the projection (VIP) threshold (VIP > 1). The false-discovery rate (FDR) values based on the two-sided P-values calculated from the nonparametric Mann-Whitney U test (FDR < 0.05) were selected, and their variations are summarized in Supplemental Tables 1 and 2. Then, according to the mean rank of the Mann-Whitney U test, when compared with Group A, all of the metabolites were found to be significantly decreased in Groups B and C.

### Characterization and functional analysis of the key metabolic pathways

**Figure 3:**
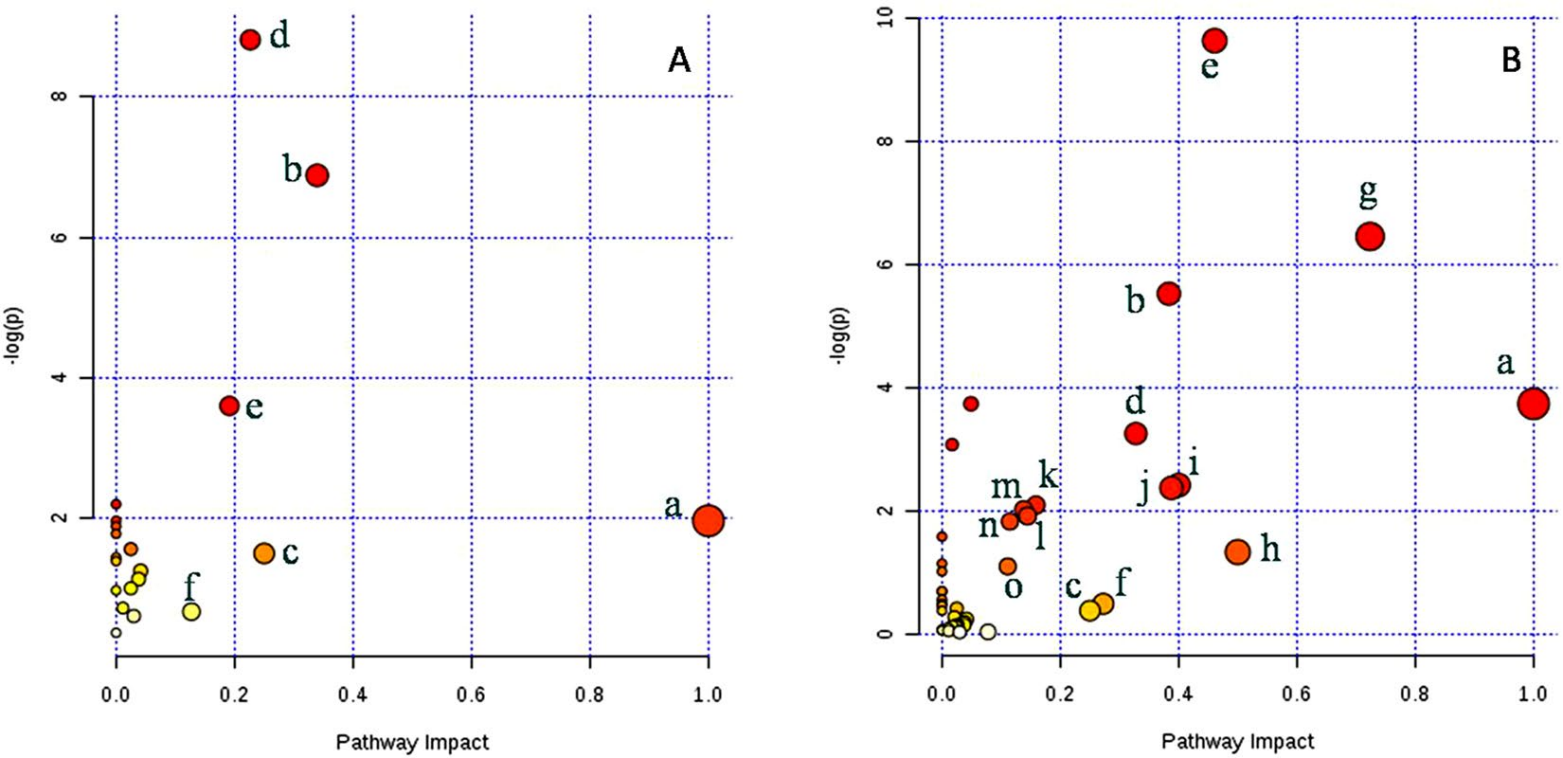
The metabolome view map of relevant metabolic pathways for change in plasma metabolic profiles. (**A**) Group A vs. Group B; (**B**) Group A vs. Group C. Light blue means the metabolite are is not in our data but used as background for enrichment analysis; grey means the metabolite is not in our data and also excluded from enrichment analysis; other colors (varying from yellow to red), means the metabolites are in our data with different levels of significance for enrichment analysis; the original p value calculated from the enrichment analysis; impact value is the pathway impact value calculated from pathway topology analysis. (a) alpha-linolenic acid; (b) glycerophospholipid metabolism; (c) pentose and glucuronate interconversions; (d) glycerolipid metabolism; (e) folate biosynthesis; (f) tyrosine metabolism; (g) one-carbon pool by folate; (h) phenylalanine, tyrosine and tryptophan biosynthesis; (i) thiamine metabolism; (j) pantothenate and CoA biosynthesis; (k) purine metabolism; (l) inositol phosphate metabolism; (m) amino sugar and nucleotide sugar metabolism; (n) pentose phosphate pathway; (o) citrate cycle (TCA cycle).

The 57 altered metabolites in Groups A and B and the 272 altered metabolites in Groups A and C were selected to carry out metabolomics pathway analysis (MetPA). The detailed results of the pathway analysis for Groups A and B and for Groups A and C are listed in Supplemental Tables 3 and 4, respectively. The relevant pathways for Groups A and B and for Groups A and C were visualized via an interactive visualization framework (Fig. 3A,B).

The metabolic pathways with impact value > 0.1 or- log (p) > 10 are considered the most relevant pathways involved in the conditions under study^10^. Hence, six metabolic pathways were selected as potential metabolic pathways for Groups A and B according to their impact value (Table 1). The detected metabolites involved in these potential metabolic pathways are summarized in Table 1. Among these pathways, three biological modules were involved in lipid metabolism, including glycerolipid metabolism, glycerophospholipid metabolism, and alpha-linolenic acid metabolism. The other three biological modules included folate biosynthesis, pentose and glucuronate interconversions, and tyrosine metabolism.

**Table 1:** The detailed results of potential metabolic pathways for Groups A vs. B.

Potential metabolic pathway	−log(p)	Impact	Relevant metabolites
Glycerolipid metabolism	8.81	0.23	TGs DGs Glycerol 3-phosphate Phosphatidic acid
Glycerophospholipid metabolism	6.88	0.34	TGs DGs Glycerol 3-phosphate Phosphatidic acid
Folate biosynthesis	3.60	0.19	2,5-diaminopyrimidine nucleoside Triphosphatetetrahydrobiopterin
Alpha-linolenic acid metabolism	1.97	1	Alpha-linolenic acid
Pentose and glucuronate interconversions	1.50	0.25	Deoxycholic acid 3-glucuronide
Tyrosine metabolism	0.68	0.13	L-dopa

**Note:–log (p)** is from the original P-value calculated from the enrichment analysis; **Impact** is the pathway impact value calculated from pathway topology analysis; **TGs** include TG(14:0/17:1(9Z)/19:1(9Z))[iso6] and TG(58:2); **DGs** include DG(14:1/22:6), DG(14:1/22:6), DG(15:0/0:0/18:0), DG(15:0/20:5), DG(16:0/14:1), DG(16:0/16:0), DG(18:1/15:0), DG(18:3/20:5), DG(20:1/14:0), DG(20:2/15:0), DG(20:2(11Z,14Z)/18:3(9Z,12Z,15Z)/0:0), DG(20:3/0:0/18:2), DG(20:3/18:1), DG(20:4/22:0), DG(20:4(5Z,8Z,11Z,14Z)/22:5(7Z,10Z,13Z,16Z,19Z)/0:0), DG(22:4/14:0), DG(22:6/14:0), DG(22:6/20:4) and DG(22:5(7Z,10Z,13Z,16Z,19Z)/15:0/0:0).

Fifteen metabolic pathways were selected as potential metabolic pathways for Groups A and C according to their impact value (Table 2). Interestingly, the fifteen metabolic pathways included the six pathways for Groups A and B. The detected metabolites involved in these potential metabolic pathways are summarized in Table 2. Among these pathways, three biological modules were involved in lipid metabolism, including glycerolipid metabolism, glycerophospholipid metabolism, and alpha-linolenic acid metabolism. Four biological modules were involved in the metabolism of the cofactors and vitamins, including folate biosynthesis, the one-carbon pool affected by folate (one-carbon cyclic metabolism by folate), thiamine metabolism, and pantothenate and CoA biosynthesis. Five biological modules were found to be involved in carbohydrate metabolism, including amino sugar and nucleotide sugar metabolism, inositol phosphate metabolism, the pentose phosphate pathway, the tricarboxylic acid cycle (TCA), and pentose and glucuronate interconversions, including the pentose phosphate pathway and the glucuronate interconversions pathway. Three biological modules were involved in AA metabolism, including phenylalanine, tyrosine, and tryptophan biosynthesis; tyrosine metabolism; and cysteine and methionine metabolism. In addition, purine metabolism (impact = 0.16) was determined to be involved in the nucleotide metabolism.

**Table 2:** The detailed results of potential metabolic pathways for Groups A vs. C.

Potential metabolic pathway	−log(p)	Impact	Relevant metabolites
Folate biosynthesis	9.63	0.46	Tetrahydrofolic acid Dihydrobiopterin 2,5-Diaminopyrimidine nucleoside triphosphate Guanosine triphosphate Folic acid Dihydroneopterin triphosphate Tetrahydrobiopterin
One-carbon pool by folate	6.45	0.72	5-Methyltetrahydrofolic acid 5,10-Methenyltetrahydrofolic acid Tetrahydrofolic acid Folic acid
Glycerophospholipid metabolism	5.52	0.38	Phosphatidylethanolamine Phosphatidylcholine LysoPC(18:1(9Z)) Glycerol 3-phosphateGlycerylphosphorylethanolamine Glycerophosphocholine
Alpha-linolenic acid metabolism	3.74	1	Phosphatidylcholine Alpha-linolenic acid Stearidonic acid
Glycerolipid metabolism	3.25	0.33	TGs DGs MGs Glycerol 3-phosphate
Thiamine metabolism	2.42	0.4	Thiamine Thiamine triphosphate
Pantothenate and CoA biosynthesis	2.37	0.39	Pantetheine 4′-phosphate D-4′-Phosphopantothenate Pantothenic acid;
Purine metabolism	2.09	0.16	ADP Deoxyadenosine Guanosine monophosphate Deoxyguanosine Guanosine triphosphate Adenosine diphosphate ribose 2’-Deoxyinosine triphosphate 5-Aminoimidazole ribonucleotide
Amino sugar and nucleotide sugar metabolism	2.02	0.14	N-Acetyl-D-glucosamine Guanosine diphosphate mannose L-Fucose Cytidine monophosphate N-acetylneuraminic acid GDP-4-Dehydro-6-deoxy-D-mannose;
Inositol phosphate metabolism	1.91	0.14	1D-Myo-inositol 1,4,5,6-tetrakisphosphate Inositol 1,3,4-trisphosphate 1D-Myo-inositol 1,3,4,6-tetrakisphosphate;
Pentose phosphate pathway	1.83	0.11	Deoxyribose 5-phosphate 6-Phosphogluconic acid Gluconolactone
Phenylalanine, tyrosine and tryptophan biosynthesis	1.33	0.5	L-dopa and L-tyrosine
Citrate cycle (TCA cycle)	1.09	0.11	Isocitric acid
Tyrosine metabolism	0.49	0.27	L-Dopa L-Tyrosine
Pentose and glucuronate interconversions	0.36	0.25	3-Methoxy-4-hydroxyphenylglycol glucuronide

**Note:–log (p)** is from the original P-value calculated from the enrichment analysis; **Impact** is the pathway impact value calculated from pathway topology analysis; **TGs** include TG(14:0/18:0/20:3), TG(15:0/14:1/15:0), TG(15:0/18:1/16:1), TG(15:0/18:3(6Z,9Z,12Z)/14:1(9Z)), TG(15:0/22:0/22:0), TG(16:0/16:1(9Z)/18:0)[iso6], TG(16:1(9Z)/14:0/16:1(9Z))[iso3], TG(18:0/18:0/22:6) and TG(22:1/18:3/18:2); **DGs** include DG(14:0/0:0/22:0), DG(14:0/16:1), DG(14:1/22:2), DG(14:1/22:6), DG(15:0/18:3), DG(15:0/20:5), DG(15:0/22:6), DG(16:0/14:1), DG(16:0/16:0), DG(16:0/24:1), DG(16:1/0:0/22:6), DG(16:1/14:1), DG(16:1/20:3), DG(18:0/14:1), DG(18:0/15:0), DG(18:0/18:0/18:3), DG(18:1/14:1), DG(18:1/15:0), DG(18:3/20:5), DG(18:4/20:4), DG(20:0/0:0/20:0), DG(20:0/22:1), DG(20:1/18:4), DG(20:2/15:0), DG(20:3/18:1), DG(20:2(11Z,14Z)/18:3(9Z,12Z,15Z)/0:0), DG(20:3/18:3), DG(20:3/20:4), DG(20:4/18:3), DG(20:4/18:4), DG(20:4/22:0), DG(20:4(5Z,8Z,11Z,14Z)/22:5(7Z,10Z,13Z,16Z,19Z)/0:0), DG(20:5/14:1), DG(20:5/15:0), DG(20:5/16:1), DG(20:5/18:4), DG(22:0/20:1), DG(22:0/22:5), DG(22:4/14:0), DG(22:5(7Z,10Z,13Z,16Z,19Z)/15:0/0:0), DG(22:5/18:2), DG(22:5/20:1), DG(22:5/20:4), DG(22:5/22:5), DG(22:6/14:0), DG(22:6/15:0), DG(22:6/18:4), DG(22:6/20:4), DG(22:6/22:5), DG(24:0/18:0), DG(24:1/0:0/18:2) and DG(24:1/22:2); MGs include MG(20:2/0:0), MG(18:1(9Z)/0:0/0:0), MG(20:3(11Z,14Z,17Z)/0:0/0:0) and MG(20:5/0:0).

## Discussion

In the cow, the embryonic period of gestation extends from conception to the end of the differentiation stage (approximately 42 days) and the foetal period extends from D 42 to parturition^11^. Although most pregnancy losses occur during the early embryonic period, the incidence of early foetal loss is increasing under intensive management systems for dairy cattle^12^. Hence, three time points (D 0, D 17 and D 45 after AI) were selected in this study to characterize the maternal metabolic response to successful pregnancy at early embryonic and foetal stages. An LC-MS metabonomics technique was devised to reveal metabolic changes of cows on D 0, D 17 and D 45. The results showed that the metabolic profiles of cows on D 17 and D 45 were significantly different from those of cows on D 0. Six metabolic pathways had changed on D 17 and D 45, and another 9 metabolic pathways had also changed on D 45. However, no significant differences existed between cows on D 17 and D 45.

Plasma triacylglycerols (TGs), diacylglycerols (DGs) and their metabolites involved glycerolipid metabolism and glycerophospholipid metabolism decreased on D 17 and D 45 of pregnancy, which may be related to changes in the use of fatty acids for energy production via fatty acid beta-oxidation. The ewe endometrial lipid abundance increased as the oestrous cycle progressed from D 3 to D 12 and then to D 15, whereas during early pregnancy, endometrial lipid concentrations decreased on D 15 of pregnancy compared with that on D 3 and D 12^13^. These findings indicate that lipids are accumulated in the uterus before insemination and then gradually consumed as a source of energy for embryo via fatty acid beta-oxidation as pregnancy progresses. Therefore, the decreases in TGs, DGs and their metabolites in the maternal plasma may reflect an increased requirement for fatty acid beta-oxidation by the uterus to provide energy for the embryo and foetus on D 17 and D 45 of pregnancy in dairy cows.

An “essential fatty acid”, alpha-linolenic acid (ALA), was decreased on D 17 and D 45 of pregnancy in this study, consistent with a woman’s essential fatty acid status during pregnancy^14^. From the physiological point of view, the most important role in maternal-foetal metabolism is performed by long chain poly-unsaturated fatty acids (LC-PUFAs), the most important of which include ALA^15–17^. ALA is also a precursor for other biologically important LC-PUFAs. Derivatives of ALA are represented by docosahexaenoic acid (DHA), which is necessary for brain development^14^, and eicosapentaenoic acid (EPA), a precursor of numerous prostanoids and leukotrienes that are essential in foetal development. Due to the lack of certain elongases and desaturases in the placenta, the biosynthesis of the most important LC-PUFAs, such as DHA or EPA, occurs in the mother and partly in the liver of the foetus^18^. Therefore, we hypothesized that ALA, from the beginning of pregnancy, may be used to synthesize most important LC-PUFAs by the mother for embryo and foetal development, causing its blood concentration to decrease on D 17 and D 45 of pregnancy.

The change in tyrosine metabolism was reflected by decreased plasma L-dopa on D 17 of pregnancy, and the change in phenylalanine, tyrosine, and tryptophan biosynthesis was reflected by decreased plasma L-dopa and L-tyrosine on D 45 of pregnancy. A decrease in maternal blood L-tyrosine has a positive effect on a successful pregnancy because a high dose of tyrosine can significantly decrease the concentration of serum progesterone to terminate early pregnancy in mice^19^. L-tyrosine is converted into L-dopa and subsequently into catecholamine *in vivo*. Catecholamine is sharply decreased in a pregnant uterus compared with a non-pregnant uterus in animals of various species, the physiological relevance of this change in the uterus is likely that the ability of the adrenergic innervation to elicit a contractile response is considerably limited^20^. Therefore, a decrease in maternal plasma L-dopa or L-tyrosine is probably responsible for a reduced catecholamine in the uterus in this study.

In this study, only plasma triphosphatetetrahydrobiopterin and 2,5-diaminopyrimidine nucleoside involved in the folate biosynthesis pathway decreased on D 17, then folic acid and more other metabolites involved in this pathway decreased on D 45. In 2010, Kwong *et al*. provided insight into the importance of maternal dietary folate/B-vitamin status during the peri-conceptional period in bovines^18^, although the supply of folic acid by the diet and the synthesis by ruminal microflora is sufficient to prevent folic acid deficiency in dairy cows and to maintain normal gestation^21^. Pregnant women are more prone to folic acid deficiency due to the sharp increase in maternal consumption caused by the maternal folic acid being transported to a foetus via the placenta^22^ together with the increase folic acid output due to changes in the maternal renal function. Therefore, a decrease in plasma metabolites in the folate biosynthesis pathway on D 17 and D 45 of pregnancy is probably due to maternal folic acid being transported to the uterus for embryo and foetal development.

Plasma metabolites involved thiamine metabolism and TCA cycles decreased on D 45 of pregnancy. Folic acid can promote thiamine absorption from the intestinal tract. Accompanied by a decrease in folate, serum retinol, pyridoxal 5′-phosphate and thiamine decrease in humans during pregnancy^23^. Thiamine is the precursor of thiamine pyrophosphate, which is an important coenzyme involved in the oxidative decarboxylation of pyruvate and alpha ketoglutaric acid in TCA cycles. Accordingly, a decrease in plasma metabolites involving thiamine metabolism and TCA cycles may be due to reduced folic acid.

A central function of folate-mediated one-carbon metabolism is to generate and transfer One-carbon units for the de novo synthesis of purines, thymidylate andremethylation of methionine^18^. Hence, a decrease in metabolites involving one-carbon metabolism, cysteine and methionine metabolism and purine metabolism on D 45 may be the consequence of reduced folic acid.

The pantothenate and CoA biosynthesis pathway may be involved in foetal development in dairy cows. We observed that pantothenic acid and their metabolites decreased on D 45 of pregnancy. PA is a part of the CoA and acyl carrier protein of fatty acid synthase in many metabolic pathways. Maternal PA deficiency during embryogenesis in rats has been reported to produce congenital malformation and to retard foetal growth^24^. The blood pantothenic acid concentrations of pregnant women shows exhibit lower levels than those in non-pregnant women in the same age classes^25^. In this the present study, the decreased maternal plasma pantothenic acid level on D 45 of pregnancy was probably due to the transfer of PA to the foetus via the uterus^24^.

The plasma metabolites involving the inositol phosphate metabolism pathway, such as inositol 1, 3,4-trisphosphate (IP_3_), on D 45 were lower than on D 0. Such changes may result from the change in progesterone concentration during the period of maternal recognition of pregnancy. Oxytocin-stimulated secretion of PGF_2a_ probably occurs via activation of the inositol phosphate (IP)/diacylglycerol second-messenger system within the endometrium^26^. The induction of oxytocin-stimulated endometrial turnover of IP between D 12 and 16 of the cycle may be involved in the luteolytic mechanism, and a conceptus-dependent endometrial block to oxytocin-stimulated turnover of IP occurs during the period of maternal recognition of pregnancy in ewes^27^. Progesterone involves the inhibition of oxytocin-stimulated turnover of IP^28^.

Maternal plasma metabolites involving pentose and glucuronate interconversion pathways decreased on D 17 of pregnancy. Subsequently, metabolites involving pentose and glucuronate interconversions, the pentose phosphate pathway, amino sugar and nucleotide metabolism and purine metabolism decreased on D 45 of pregnancy. These metabolic pathways are associated with nucleic acid biosynthesis. Accordingly, maternal plasma nucleic acids might be diminished because they may be needed in the uterus as a mechanism to generate energy for embryo development without using glucogenic precursors.

In conclusion, this study presents the first integrated analyses of metabonomics and pathway analysis in dairy cows during early pregnancy. Our data show that the obvious changes in plasma metabolic profiles occur in dairy cows at early embryonic and foetal stages, which is reflected by variations in the levels of six metabolic pathways that are obvious at early embryonic stages and fifteen metabolic pathways at early foetal stages. Clearly, the combination of metabonomics and pathway analysis generates rich biochemical insight into the possible biological modules related to embryo and foetal development. However, a further targeted study in uterus tissue and maternal blood is required to test the metabolic changes.

## Materials and Methods

### Study subjects and plasma collection

Holstein multiparous cows from a dairy farm (Yinchuan City, China) were used for plasma sampling. The study was performed according to the international, national and institutional rules considering animal experiments, clinical studies and biodiversity rights and approved by the Animal Ethics Committee of Ningxia University (permit number: 0281/2016).

Twelve plasma samples from dairy cows on D 0 at the time of AI were referred to as Group A. Twelve plasma samples were collected and retained from dairy cows 17 days after AI, eleven of which were selected as Group B because the corresponding dairy cows were later confirmed by transrectal ultrasonography to have been successfully impregnated on D 45 of pregnancy. Fourteen plasma samples (referred to as Group C) were collected 45 days after timed AI from dairy cows that were confirmed by transrectal ultrasonography to have been successfully impregnated. All dairy cows were in their second lactation and selected for this study according to their body weight, body condition score, and milk yield to attenuate possible effects of different conditions and merit. All cows were oestrus synchronized by two injections of prostaglandin F2α 14 days apart. Each group of cows was inseminated on the same day.

Morning fasting blood samples were collected from the tail vein using a heparin (10 IU/mL) anticoagulant blood tube. The plasma samples were immediately prepared by centrifugation at 3,000 rpm for 10 minutes and then stored at −80 °C until used for the metabonomics analysis.

### Sample preparation and pretreatment

Prior to the LC/MS analysis, the plasma samples were thawed at room temperature for 15 minutes and vortexed vigorously for five seconds. Next, 300 μL of HPLC-grade methanol (Merck, Germany) was added to 100 μL of the plasma samples, and they were then vigorously vortexed again for another 30 seconds. The mixtures were centrifuged at 12,000 rpm for 15 minutes at 4 °C. Supernatant (200 μL) was transferred to a high-performance liquid chromatography (HPLC) auto-sampler injection vial for the LC/MS analysis. To ensure the stability and repeatability of the HPLC-QTOF systems, pooled quality control (QC) samples were prepared from 20 μL of each sample, and these were then staggered through the other samples (after every five).

### HPLC-QTOF/MS analysis

HPLC-QTOF/MS analysis was performed on a 4 μL aliquot of the pretreated plasma samples using a C18 (Agilent, 2.1 mm × 100 mm × 1.8 μm) column held at 40 °C using the Agilent 1290 Infinity LC System (Agilent Technologies). The mobile phase was made up of A (water with 0.1% formic acid) and B (acetonitrile with 0.1% formic acid). The metabolites were eluted with a gradient of 5% B for 0 to one minute; 5% to 20% B for one to six minutes; 20% to 50% B for six to nine minutes; 50% to 95% B for nine to 13 minutes; and then maintained at 95% B for 13 to 15 minutes. The flow rate was 0.4 mL/min, and the samples were maintained at 4 °C during the analysis.

In this study, the mass spectrometry was performed using an Agilent 6530 UHD and an Accurate-Mass QTOF (Agilent Technologies) equipped with an electrospray ionization source operating in either positive or negative ion mode. The source temperature was set at 100 °C with a cone gas flow rate of 50 L/h. The dissolving gas temperature was 350 °C with a flow rate of 600 L/h in positive ion mode (ES^+^), and at 300 °C, the flow rate was 700 L/h in negative ion mode (ES^−^). The capillary voltages were set at 4 kV ES^+^ and 3.5 kV in ES^−^. The sampling cone voltage was set at 35 kV in ES^+^ and 50 kV in ES^−^. The extraction cone voltage was set at 4 V in ES^+^ and ES^−^. The centroid data were collected from 50 m/z to 1,000 m/z with a scan time of 0.03 seconds and an inter-scan delay of 0.02 seconds. All of the analyses were acquired using a lock spray feature to ensure accuracy and reproducibility, and leucine-enkephalin was used as the lock mass (m/z 556.2771 in ES^+^, and 554.2615 in ES^−^)^29^.

### Data preprocessing and annotation

The raw HPLC-QTOF/MS ESI data were converted to mz format data using a Mass Profiler (Agilent). The files were then imported to a XCMS package (R program) for preprocessing, which included nonlinear retention time (RT) alignment and matched filtration, as well as peak detection and matching^30^.

Finally, the output data were manually searched and edited using EXCEL 2007 software, which included the elimination of impurity peaks and duplicate identifications. The final results were changed into a 2D data matrix, including the variance (Rt/mz), observed quantity (code of each plasma sample), and peak intensities.

### Statistical analysis

The two data sets that resulted from the HPLC-Q/TOF/MS ES^+^ and ES^−^ were mean centred, unit variance scaled, and combined prior to a multivariate statistical analysis using SIMCA-p 13.0 software. The unsupervised method (PCA) and the supervised method (OPLS-DA) were both employed to reveal the metabolic changes between groups. The validity of the model was certified via a 7-fold cross-validation method^31^ along with a permutation test method^32^. The goodness-of-fit parameters for the OPLS model (R^2^X, R^2^Y, and Q^2^Y) were then calculated and were determined to vary from 0 to 1. R^2^X and R^2^Y represented the fraction of the variance of the x and y variables explained by the model, respectively. Q^2^Y described the predictive performance of the model. The corresponding VIP values were also calculated in the OPLS-DA model. On the basis of a VIP threshold of 1, from the 7-fold cross-validated OPLS-DA model, a number of metabolites identified as being different among the A, B, and C groups, were described. The metabolites that significantly differed between groups were determined using a nonparametric Mann-Whitney U test (SPSS version 13.0), with the critical P-value set at 0.05. The raw P-values were adjusted using a Benjamini and Hochberg procedure (BH method)^33^. A FDR control was executed to correct for multiple comparisons. The adjusted P-values that were less than the desired FDR (5%) were considered to be significant.

### Identification of the plasma biomarkers

For the identification of potential biomarkers, biochemical databases, including HMDB (http://www.hmdb.ca/), KEGG (http://www.genome.jp/kegg/), METLIN (http://metlin.scripps.edu/), Bovine Metabolome Database (http://www.cowmetdb.ca/), SMPD (http://www.smpdb.ca/), and MASSBANK (http://www.massbank.jp/), were used for comparison based on mass within 30 Da, with the fragment information obtained from the HPLC-QTOF/MS. The list of potential metabolites following the database matching is shown in Tables S1 and S2.

### Related pathway characterization

MetPA was used to expand the characterization of the metabolomic analyses and to clearly understand the system-level effects of the variation in metabolites^10^. The metabolites were imported into a Metaboanalyst 3.0 (http://www.metaboanalyst.ca/) to generate the metabolome view, which integrated the pathway enrichment analysis and the pathway topology analysis. *Bos taurus* (cow) was selected as the model organism. An over-representation analysis (ORA)^34^ was applied for the functional enrichment analysis. The ORA was implemented using hypergeometric testing in order to evaluate whether a particular metabolite set was represented more than expected by chance within the metabolite sets^35^. The pathway topological analysis was based on the relative betweenness and out-of-degree centrality measures of a metabolite in a given metabolic network and for the purpose of calculating the metabolite’s importance^36^. Potential targets were selected either according to the P-values from the pathway enrichment analysis or based on the impact values from the pathway topology analysis^37^. The impact value threshold was set as 0.10, and the negative-log P-value threshold was set as 10. Each different metabolite was cross-listed with the pathways. The top-level altered pathways were then identified and constructed in accordance with the potential functional analysis.

## Electronic supplementary material

Supplemental figure 1

Supplemental table 1

Supplemental table 2

Supplemental table 3

Supplemental table 4
