# Supplementary material for: Metabolomics and pathway analyses to characterize metabolic alterations in pregnant dairy cows on D 17 and D 45 after AI
Source: Sci Rep. 2018 Apr 13;8:5973. doi: 10.1038/s41598-018-23983-2 (PMC5899158; doi:10.1038/s41598-018-23983-2)
Supplement: Supplementary file 1 — Supplemental figure 1 [file 41598_2018_23983_MOESM1_ESM.docx]

Y. S. Guo1, J.Z.Tao1: Metabolomics and pathway analyses to characterize metabolic alterations in pregnant dairy cows on D 17 and D 45 after AI
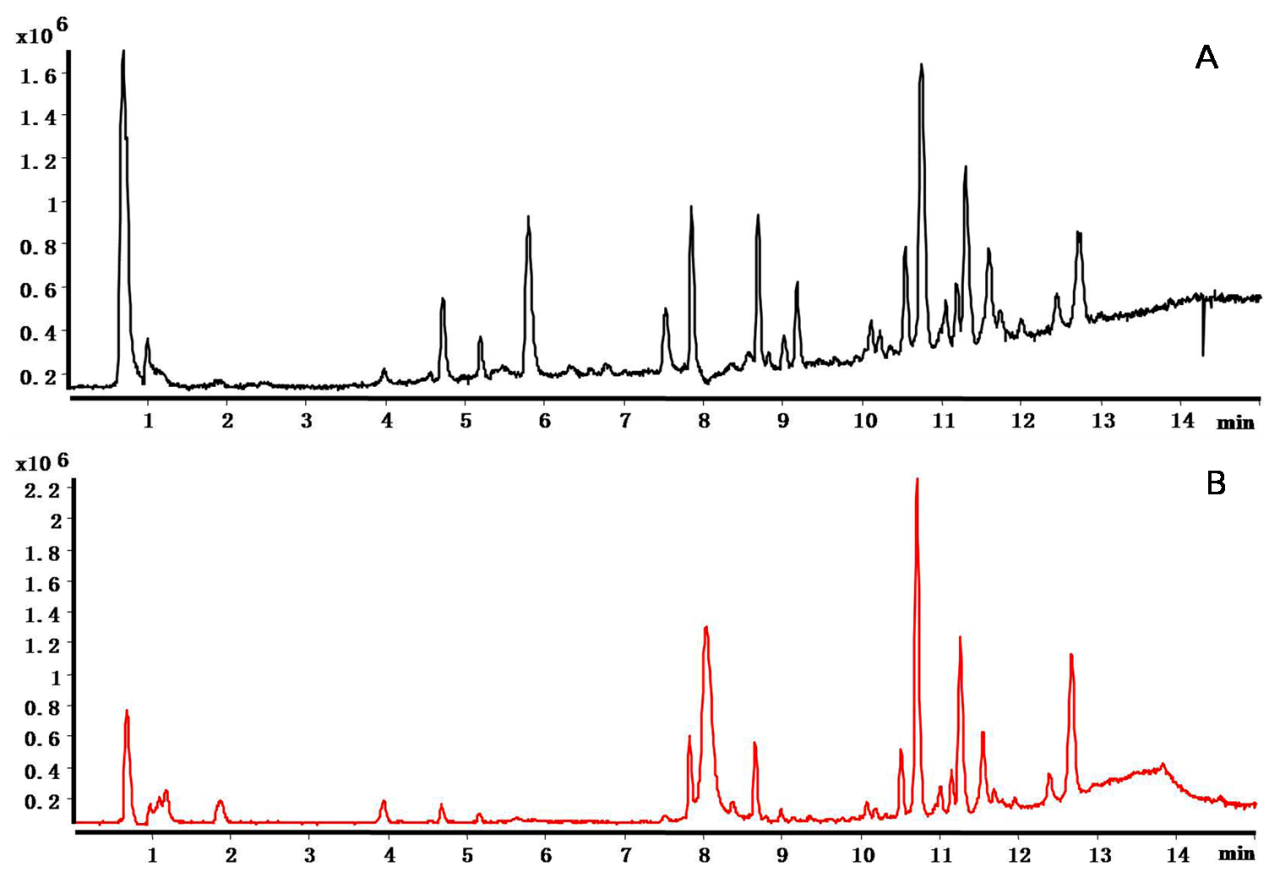


**Supplemental Figure 1:** Total ion chromatograms (TIC) of typical LC-MS plasma sample (A:-ESI, B: +ESI ). source temperature was set at 100°C, with a cone gas flow rate of 50 L/h. The dissolving gas temperature was 350°C, with a flow rate of 600 L/h in positive ion mode (ES+), and at 300°C, the flow rate was 700 L/h in negative ion mode (ES-). The capillary voltages were set at 4 kV ES+ and 3.5 kV in ES-. The sampling cone voltage was set at 35 kV in ES+, and 50 kV in ES-. The extraction cone voltage was set at 4 V in ES+ and ES-. The centroid data were collected from 50 to 1,000 m/z, with a scan time of 0.03 seconds, and an inter-scan delay of 0.02 second. All of the analyses were acquired using a lock spray feature to ensure accuracy and reproducibility, and Leucine-enkephalin was used as the lock mass (m/z 556.2771 in ES+, and 554.2615 in ES-)
